# Biomarkers in Vasculitides of the Nervous System

**DOI:** 10.3389/fneur.2019.00591

**Published:** 2019-06-06

**Authors:** Daniel Strunk, Antje Schmidt-Pogoda, Carolin Beuker, Lennart S. Milles, Catharina Korsukewitz, Sven G. Meuth, Jens Minnerup

**Affiliations:** Department of Neurology, Institute for Translational Neurology, University of Münster, Münster, Germany

**Keywords:** PACNS, Primary systemic vasculitides, biomarkers, inflammation, differential diagnoses

## Abstract

Besides being affected by the rare and severe primary angiitis of the central nervous system (PACNS) the nervous system is also affected by primary systemic vasculitides (PSV). In contrast to PACNS, PSV affect not only the central but also the peripheral nervous system, resulting in a large array of potential symptoms. Given the high burden of disease, difficulties in distinguishing between differential diagnoses, and incomplete pathophysiological insights, there is an urgent need for additional precise diagnostic tools to enable an earlier diagnosis and initiation of effective treatments. Methods available to date, such as inflammatory markers, antibodies, cerebrospinal fluid (CSF) analysis, imaging, and biopsy, turn out to be insufficient to meet all current challenges. We highlight the use of biomarkers as an approach to extend current knowledge and, ultimately, improve patient management. Biomarkers are considered to be useful for disease diagnosis and monitoring, for predicting response to treatment, and for prognosis in clinical practice, as well as for establishing outcome parameters in clinical trials. In this article, we review the recent literature on biomarkers which have been applied in the context of different types of nervous system vasculitides including PACNS, giant-cell arteritis, Takayasu's arteritis, polyarteritis nodosa, ANCA (anti-neutrophil cytoplasm antibody)-associated vasculitides, cryoglobulinemic vasculitis, IgA vasculitis, and Behçet's disease. Overall, the majority of biomarkers is not specific for vasculitides of the nervous system.

## Introduction

Primary angiitis of the central nervous system (PACNS) is a rare and often devastating disease with high morbidity and mortality. Major clinical manifestations include ischemic and hemorrhagic stroke, headache and encephalopathy (1). In addition to PACNS, the nervous system may be also affected by primary systemic vasculitides (PSV), which manifest primarily in the context of vasculitides of small and medium sized vessels, e.g., in ANCA-associated vasculitides and polyarteritis nodosa. Due to the severity of nervous system involvement, aggressive immunosuppressive treatments, e.g., high-dose glucocorticoids and cyclophosphamide, are frequently required for remission induction in both, PACNS and PSV. Nonetheless, chronic neuronal damage and persisting symptoms are frequent, even after early immunosuppressive treatment initiation (1).

With respect to the high disease burden there is an urgent need for additional precise diagnostic tools enabling an early diagnosis and treatment initiation. The use of biomarkers may emerge as a valuable approach to overcome these problems. The term “biomarker” is based on the two words “biological” and “marker.” Biomarkers can be extracted from different kinds of body fluids and tissues, and are applied as surrogate parameters for various medical conditions (2, 3).

This review aims to give a concise overview of current areas of application for biomarkers with regard to pathogenesis, clinical manifestation, and management of PACNS and those PSV with nervous system involvement. Although biomarkers derived from biopsy specimens are of unquestionable value, this review puts special emphasis on biomarkers derived from body fluids, because biomarkers that can be isolated from body fluids are more likely to be integrated in daily clinical practice (3).

## Biomarkers in primary angiitis of the central nervous system (PACNS)

PACNS is an important cause of stroke and is difficult to differentiate from other conditions that also result in stroke (4). Men are affected twice as often as women. The mean age at disease onset is 50 years (5). Symptoms of PACNS are diverse and not specific. Among them are, in particular, headache, altered cognition, and focal neurologic deficits such as hemiparesis, hemihypesthesia, ataxia, aphasia, dysarthria, and visual disturbances (6). Further typical clinical manifestations are seizures and encephalopathy. The gold standard for the diagnosis of PACNS is a biopsy of brain parenchyma and leptomeninges. Due to possible sampling errors, a negative result does not necessarily mean that PACNS can be ruled out, though (7). Further examinations, including magnetic resonance imaging (MRI), magnetic resonance angiography (MRA), digital subtraction angiography (DSA), or cerebrospinal fluid (CSF) analysis exhibit a rather high degree of sensitivity whereas specificity assumes low values (8). Well-known markers of inflammation and autoimmunity, such as C-reactive protein (CRP), erythrocyte sedimentation rate (ESR), rheumatic antibodies (ANA, dsDNA, ENA, ANCA), and oligoclonal bands do not play a decisive role in PACNS (9).

Our own group retrospectively analyzed the composition of CSF immune cells in patients with PACNS in comparison with sex- and age-matched patients with ischemic stroke, multiple sclerosis, and somatoform disorders by means of multi-parameter flow cytometry (10). PACNS patients were shown to have higher CSF leukocyte counts than controls (10). Some individuals exhibited a shift toward NK (natural killer) or B cells while proportions of T cell subsets remained unmodified. In other patients, we detected higher numbers of plasma cells and an immunoglobulin synthesis within the central nervous system (10). Altogether, characteristics of the intrathecal immune-cell profile were heterogenous in PACNS patients in this study (10).

Ruland et al. employed ion mobility mass spectrometry for unbiased proteomic profiling to further elucidate the pathophysiologic principles and potential biomarkers of PACNS, and identified fourteen proteins from neuronal structures that might be of importance, among others amyloid—beta A4 protein (APP) (11). Amyloid-beta proteins are metal chelators which reduce metal. They are said to activate mononuclear cells in the central nervous systems and evoke inflammatory processes. Both, APP or its fission product beta-amyloid, respectively, are known to be involved in other diseases of the vasculature of the central nervous system, e.g., Amyloid Beta-Related Angiitis (ABRA) (9, 11). This is why the authors highlight the possibility of inflammatory and degenerative processes being intertwined in PACNS (11).

Thom et al. evaluated the potential role of the proinflammatory cytokine interleukin-17 (IL-17), measured in the CSF by flow cytometry, as a biomarker for cerebral vasculitis in patients with stroke. As a major finding, Thom et al. found a marked and persisting increase in IL-17 production in individuals with PACNS compared to patients with non-inflammatory neurological diseases (12). Another study supports the concept of CD4^+^ T cells being of major importance in vasculitic conditions by detecting an elevated percentage of this cell type in the CSF of individuals with amyloid-ß related cerebral angiitis (13), thus suggesting intrathecal CD4^+^ T cells as a possible biomarker in PACNS.

The amount of circulating von Willebrand factor antigen (vWF) seems to correlate with a clinical global assessment of disease activity in childhood PACNS (14). vWF is a glycoprotein important for platelet aggregation and the protection of coagulation factor VIII from proteolysis (15). vWF is released by endothelial cells after vascular injury. Consequently an increase in vFW levels may indicate inflammatory processes of the vasculature (14). vWF seems to be an informative indicator of active inflammation in infants but not in adults due to increasing vWF levels with age (14). In Cellucci's study, increased vWF antigen levels were detected at the timepoint of diagnosis in 65% of children with PACNS and showed a significant decrease after treatment initiation (14). The authors found that elevated values of vWF antigen at the timepoint of diagnosis might be indicative of a less active cPACNS after 12 months of course of disease (14). Therefore, vWF antigen represents a promising biomarker of disease activity in children. One of the study's limitations is that results have been derived from experiences at a single medical center in Canada. Consequently its findings cannot be generalized, the more so as adult patients were not examined (14). To overcome these limitations, further investigations in children and adults should be carried out.

In contrast to vWF, the acute phase reactants C-reactive protein (CRP) and erythrocyte sedimentation rate (ESR) did not show a correlation with disease activity, which is not surprising, given that their levels are known to increase in response to many kinds of inflammatory states not necessarily linked to the vasculature (14).

Taking the above mentioned limitations into consideration, none of these potential biomarkers is sufficiently tested and validated in adult patients with PACNS. For a summary of biomarkers, see Tables 1, 2.

**Table 1:** Summary of traditional and already sufficiently tested biomarkers and their meaning in vasculitides with neurological manifestations.

**Type of vasculitis**	**Biomarker**	**Meaning**
Primary angiitis of the central nervous system	–	–
Giant-cell arteritis	CRP, ESR	Inflammatory markers, diagnosis, monitoring disease activity
	IL-6	Disease activity
Takayasu's arteritis	IL-6	Disease state and activity
Polyarteritis nodosa	–	–
ANCA (anti-neutrophil cytoplasm antibody)-associated vasculitides	CRP to serum albumin ratio	All-cause mortality
	Delta neutrophil index	Correlation with Birmingham vasculitis activity score; Predicting course of disease/relapses
	PR3-ANCA, MPO-ANCA	Diagnosis, Monitoring and predicting course of disease
Granulomatosis with polyangiitis	CRP, ESR, anti-PR3, and anti-MPO titers	Disease activity; Predicting flares
	B cell reconstitution and ANCA-PR3 level	Predicting relapse in patients treated with rituximab
Microscopic polyangiitis	–	–
Eosinophilic granulomatosis with polyangiitis	–	–
Cryoglobulinemic vasculitis	–	–
Henoch-Schönlein purpura	Serum biomarker index	Diagnosis
Behçet's disease	HLA B51	Diagnosis

*vWF, Von Willebrand factor; CSF, Cerebrospinal fluid; IL, Interleukin; CD, Cluster of differentiation; NK cells, Natural killer cells; CRP, C-reactive protein; ESR, Erythrocyte sedimentation rate; SGTB, Small Glutamine Rich Tetratricopeptide Repeat Containing Beta; FCGR3A, Fc Fragment Of IgG Receptor IIIa; CCL, chemokine (C-C motif) ligand; sIL-6R, soluble IL-6 receptor; MMP, Matrix-metalloprotease; FCGR2A/FCGR3A and RPS9/LILRB3 and PSMG1, Gene loci; HLA, Human leukocyte antigen; MPO, myeloperoxidase; PR3, Proteinase-3; CXCL, C-X-C motif chemokine; Ig, Immunoglobulin; TARC, Chemokine (C-C motif) ligand 17 (CCL17); TNF, tumor necrosis factor; HCV, Hepatitis C virus; RNA, Ribonucleic acid*.

**Table 2:** Summary of biomarkers of potential clinical value in the future and their area of application in vasculitides with neurological manifestations.

**Type of vasculitis**	**Biomarker**	**Meaning**
Primary angiitis of the central nervous system	Circulating vWF antigen	Disease activity in children
	CSF IL-17; Increased CD 4 T cells in the CSF	Diagnosis
	CSF pleocytosis; Shift toward NK or B cells; Intrathecal immunoglobulin synthesis; Plasma cells	Diagnosis; Differentiating subtypes of PACNS
Giant-cell arteritis	Pentraxin 3	Acute ischemia of the optic nerve; Disease duration <6 months
	Anti-ferritin antibodies	Diagnosis in untreated patients
	Expression of SGTB and FCGR3A	Confirmation of diagnosis; Predicting course of disease
	Serum Osteopontin	Disease activity and relapse; Disease activity in tocilizumab-treated patients
Takayasu's arteritis	Anti-ferritin antibodies	Diagnosis
	IL-8, IL-18, CCL 2, CCL 5	Diagnosis; Disease activity
	sIL-6R, MMP-1,−3, and−9, soluble receptor for advanced glycation and products, Pentraxin 3	Disease activity; Surrogate parameter of arterial inflammation and progression of vascular involvement, i.e., marker of actual arteritis (PTX-3)
	Metabolites N-acetyl glycoprotein and glutamate	Disease activity; Guide to therapy
	Plasma ghrelin level; Leptin/Ghrelin ratio	Monitoring disease activity and creating treatment strategies
	Neutrophil gelatinase-associated lipocalin	Response to treatment with glucocorticoids and tumor necrosis factor-alpha inhibitors
	Anti-endothelial cell antibodies; Antibodies against Annexin V	Disease activity; Management of TA
	Circulating endothelial progenitor cells; Vascular endothelial growth factor	Disease activity
	Serum myeloid-related protein 8/14	Diagnosis; Disease activity; Prognostic biomarker
	FCGR2A/FCGR3A, IL12B, IL6, RPS9/LILRB3, and a locus on chromosome 21 near PSMG1	Non-HLA susceptibility loci
	Serum amyloid A	Disease activity; Treatment response
Polyarteritis nodosa	Vascular endothelial growth factor	Vasculitic neuropathy
ANCA (anti-neutrophil cytoplasm antibody)-associated vasculitides	Level of activated (CD19+/CD38+) B cells; CD 25+ regulatory B cells	Disease activity
	CD8+ T cell expression	Prognosis
	Tissue inhibitor of metalloproteinase 1	Distinguishing between mildly active AAV and remission; Distinguishing active AAV and infectious disease
	Epigenetic modifications of gene loci Proteinase 3 and MPO	Correlation with disease activity, PR 3 and MPO expression
Granulomatosis with polyangiitis	MMP-3, Tissue inhibitor of metalloproteinase 1, CXCL 13	Inflammation, angiogenesis, tissue damage and repair
	Soluble receptor for advanced glycation end products	Cumulative burden of granulomatous inflammation/Disease activity
Microscopic polyangiitis	Carbonic anhydrase III antibodies	Diagnosis in patients without ANCA; Disease activity
Eosinophilic granulomatosis with polyangiitis	Eotaxin-3, IgG4, CCL17/TARC	Diagnosis; Disease activity; Differentiating from other diseases characterized by vasculitis or eosinophilia
Cryoglobulinemic vasculitis	WA B cells	Marker for the development of CV in asymptomatic patients with HCV infection
	IL-6, CXCL10, TNF-alpha serum levels	Distinguishing HCV hepatitis with or without cryoglobulinemia; Active vasculitis (Increase of CXCL10 levels)
Henoch-Schönlein purpura	White blood cells, CRP, IL-6, serum amyloid A, serum biomarker index	Diagnosis
	Fecal secondary colonic bile acids, deoxycholic acid and lithocholic acid	Diagnosis
Behçet's disease	Tyrosine-protein phosphatase non-receptor type 4, threonine synthase-like 2, and ß-actin	Diagnosis
	Serum Amyloid A	Predicting major organ involvement/ ocular disease relapse; Disease activity
	Anti-Tubulin-α-1c antibodies	Diagnosis; Degree of inflammation; Disease activity; Predicting the course/certain manifestations of BD
	Urine and serum derived metabolites	Diagnosis
	Platelet to lymphocyte ratio, Lymphocytes to monocytes ratio	Disease activity; Differentiating BD from differential diagnoses
	Tissue factor-expressing microparticles	Diagnosis; Estimating the risk of thrombosis

*vWF, Von Willebrand factor; CSF, Cerebrospinal fluid; IL, Interleukin; CD, Cluster of differentiation; NK cells, Natural killer cells; CRP, C-reactive protein; ESR, Erythrocyte sedimentation rate; SGTB, Small Glutamine Rich Tetratricopeptide Repeat Containing Beta; FCGR3A, Fc Fragment Of IgG Receptor IIIa; CCL, chemokine (C-C motif) ligand; sIL-6R, soluble IL-6 receptor; MMP, Matrix-metalloprotease; FCGR2A/FCGR3A and RPS9/LILRB3 and PSMG1, Gene loci; HLA, Human leukocyte antigen; MPO, myeloperoxidase; PR3, Proteinase-3; CXCL, C-X-C motif chemokine; Ig, Immunoglobulin; TARC, Chemokine (C-C motif) ligand 17 (CCL17); TNF, tumor necrosis factor; HCV, Hepatitis C virus; RNA, Ribonucleic acid*.

## Biomarkers in primary systemic vasculitides

The biomarkers currently used for PSV are well-known and were established before the use of the term “biomarker.” The majority of such markers are highly useful for diagnosing systemic vasculitis. Among others, antibodies to proteinase-3 (PR3) and myeloperoxidase (MPO) are highly specific for ANCA (anti-neutrophil cytoplasm antibody)-associated vasculitides. ANCA is an autoantibody against endogenous neutrophilic leukocytes. According to their target antigen they can be divided into c- (anti-PR3, elevated in tuberculosis, HIV infection, uveitis, amoebiasis, and cystic fibrosis with superinfection), p- (anti-MPO, of importance to microscopic polyangiitis, eosinophilic granulomatosis with polyangiitis, and pauci-immunen glomerulonephritis), and a-ANCA (Crohn's disease, autoimmune hepatitis) (16). The identification of ANCA is not essential for the diagnosis: About 10–20% of patients with granulomatosis with polyangiitis or microscopic polyangiitis and about 60% of patients with eosinophilic granulomatosis with polyangiitis (EPGA), previously known as Churg-Strauss syndrome, are ANCA negative (17). Erythrocyte sedimentation rate (ESR) and C-reactive protein (CRP), the most common laboratory signs of inflammation in general, are usually considered being helpful in identifying active vasculitis, especially in untreated patients, or ruling out this kind of disease. Nonetheless, all of these markers are much less useful for assessing disease activity in patients with established diagnoses. In the following, this and other aspects will be addressed in the context of the most relevant vasculitides, introducing each section with a brief engram of the respective disease.

## Large Vessel Vasculitis

### Giant-Cell Arteritis (GCA)

Giant-cell arteritis (GCA) most commonly involves vessels of large and medium size and is characterized by a granulomatous inflammatory process affecting the aorta and its main branches, predominantly the extracranial branches of the carotid artery, first of all the temporal artery (18). It is the most frequent type of vasculitis in the Western world, with women being affected more often than men. The annual incidence of GCA amounts to 20–40 per million. The disease rather affects older people, which is also reflected by the classification criteria defined by the American College of Rheumatology (19). The disorder may coexist with polymyalgia rheumatica, whose hallmarks are pain and stiffness in muscles of the pelvis and/or shoulder. Clinical manifestation of GCA comprises visual disturbances that can be temporary (Amaurosis fugax) or permanent in form of a persisting loss of vision, usually in one eye first (20, 21). Further symptoms may consist of headache, pain over the temples, jaw claudication, and a high degree of sensitivity of the temporal artery, which is prominent in many cases. Not only because of increased stroke risk, especially in the vertebrobasilar circulatory system, GCA is considered a serious disease.

Pentraxin proteins (PTX), which are involved in acute immunological responses, are under investigation as potential biomarkers of GCA. Pentraxin-3 (PTX-3), for instance, which originates from endothelial cells, smooth muscle cells and leukocytes, was found to be markedly increased in GCA patients with acute ischemia of the optic nerve compared to both, GCA cases without ischemic signs and healthy controls (22). Most interestingly, PTX-3 showed poor correlation with ESR and CRP, hinting at the potential benefit of using a combination of markers for a laboratory assessment of GCA. Generally speaking, the production of PTX3 is said to be a characteristic of damages of the vasculature (23, 24). Being synthesized at the site of vascular injury, its physiological role consists in averting further damage of the affected vessels (23, 25–27). Additional investigations are required to elucidate in how far structural modifications of affected vessels are due to the locally synthesized PTX-3 (23, 28). In the past, diagnosing GCA was sometimes hampered by low values of ESR, especially when glucocorticoids had already been administered. The informative value of biopsies is known to be significantly reduced in this constellation as well. Given that no dependency of PTX-3 levels on the respective glucocorticoid dosage was found, elevated PTX-3 values might facilitate diagnosing GCA in this context (29). A prospective study would be helpful to proof the assumption that PTX-3 levels might be of use in evaluating different types of optic nerve ischemia, i.e., distinguishing (non-) vasculitic forms (23, 30).

In addition to PTX-3, levels of IL-6 and soluble IL-6 receptor (sIL-6R) were shown to be elevated in patients with GCA (31). Il-6 is a pro-inflammatory cytokine of importance in innate immunity serving as a link between innate and specific immune response. IL-6 binds to IL-6-receptors on hepatocytes and leukocytes and soluble IL-6-receptors resulting in the initiation of immunological pathways. Finally acute-phase-reactants are activated. In particular, IL-6 levels were significantly elevated in patients with active disease, whereas sIL-6R levels turned out to be significantly higher irrespective of disease activity. Therefore, IL-6 is considered as potential biomarker for monitoring disease activity. Most importantly, the IL-6 pathway has also emerged as a promising therapeutic target in GCA patients (32). The therapeutic efficacy of IL-6 receptor alpha inhibitor Tocilizumab in the treatment of GCA patients highlights the importance of IL-6 in GCA pathophysiology.

Baerlecken et al. analyzed anti-ferritin antibodies in GCA patients in various stages of the disease. The highest levels of anti-ferritin antibodies were found in untreated individuals with a concomittant polymyalgia rheumatica, whereas treated patients and patients with inactive stages of the disease had lower levels of anti-ferritin antibodies (33, 34). Therefore, these antibodies could be useful as a diagnostic marker.

De Smit et al. suggest new ways to confirm the diagnosis of GCA and to predict the course of disease (35). By means of transcriptional profiling of T-lymphocytes, 4,031 genes in CD4^+^ and CD8^+^ cells were identified that show distinct expression patterns in GCA patients with an active disease (35). Four transcripts in CD8^+^ cells and 179 in CD4^+^ cells were identified which were characterized by alternating expression characteristics over 12 months (35). In CD8^+^ cells transcripts of SGTB [Small glutamine-rich tetratricopeptide repeat (TPR)-containing, β], playing a role in neuronal apoptosis, and FCGR (Fc gamma receptor) 3A, linked to Takayasu's arteritis maintained their altered expression pattern (35). The authors report a connection between genes and clinical aspects in the acute phase, offering the potential to predict prospective disease activity and tailor further therapies (35). The underlying mechanisms linking gene expression to actual symptoms and course of disease and the definite informative values of the aforementioned aspects in terms of a potential biomarker is to be further elucidated (35).

Prieto-González et al. reported that serum osteopontin (sOPN) might be useful in assessing and predicting disease activity in GCA (36). The glycoprotein osteopontin is of importance in the development and specialization of immune cells, inflammatory processes and remodeling in different kinds of tissues (36). Serum osteopontin concentration was significantly elevated in individuals with active disease compared to controls, whereas a significant decline was found as soon as patients entered disease remission (37). Serum osteopontin turned out to correlate with serum IL-6. Additionally, baseline serum osteopontin concentrations were significantly higher in relapsing patients than in non-relapsing patients (37).

Burja et al. investigated existing evidence on the usefulness of already known (potential) biomarkers in GCA by means of a meta-analysis. The results of this meta-analysis highlight the fact that the majority of potential biomarkers in GCA might add valuable information in understanding, diagnosing or treating the disease, but are still not suitable to be integrated in routine clinical use (38). IL-6, CRP, and ESR should, not only because of the meta-analysis by Burja et al., be considered as the best available biomarkers in GCA (for a summary of biomarkers, see Tables 1, 2).

### Takayasu's Arteritis (TA)

The prevalence of Takayasu's arteritis (TA) is lower than the prevalence of GCA. Pathophysiologically, the aorta and its main branches are affected by a granulomatous inflammatory process with massive intimal fibrosis and vascular narrowing. Takayasu's arteriitis most commonly affects young or middle-aged women of Asian descent (39). Clinical signs may be rather unspecific for several years, comprising fever, fatigue, and joint pains. Circulatory disturbances may lead to complaints in different regions of the body, e.g., in form of Raynaud's phenomenon or angina pectoris. Frequent symptoms of TA include intermittent claudication of the arms or legs, as well as a difference in pulse or blood pressure between the right and left arms or right and left legs (40). Possible neurological symptoms are transitory ischemic attacks (TIA), stroke, syncope, and posterior reversible encephalopathy syndrome (PRES) (41). Stenosis of the renal arteries is capable of causing arterial hypertension with subsequent blood pressure crises and diverse neurological consequences. Furthermore, seizures as a result of stroke or blood pressure crisis, headache, vertigo, and visual disturbances represent additional symptoms of TA. A well-known genetic susceptibility locus for TA is the human leukocyte antigen (HLA) allele HLA-B 52 (4).

Patients with TA usually exhibit an elevation of ESR and CRP. The presence of anti-ferritin antibodies has been described by Große et al. in 62% of patients with TA, compared to 0% in healthy controls and up to 92% in GCA (42). In so far there is a similarity to patients with GCA/PMR (42). Alibaz-Oner et al. found a stronger increase of IL-6, IL-8, and IL-18 in TA patients in comparison with controls, and an association of IL-18 levels with disease activity (43–46). Interleukin-8 (IL-8) is an inflammatory mediator which attracts neutrophils to the site of inflammation. Interleukin-18 (IL-18), a proinflammatory cytokine, induces the release of Interferon (IFN) γ that, in turn, plays a role in the stimulation of macrophages. It is assumed that these cytokines contribute to the emergence of vasculitic lesions in TA (45). The role of IL-6 and also sIL-6R was confirmed by Pulsatelli et al., who reported that the levels of these markers were significantly altered in TA patients compared to healthy controls (47). IL-6 levels were markedly elevated in patients with TA irrespective of disease phase, whereas a significant increase in sIL-6R levels was only detected in individuals with active disease. Longitudinal analysis demonstrated that a significant increase in sIL-6R levels was only apparent at baseline (47). Consequently, sIL-6R is suggested to mirror disease activity in TA (48). Additional research on cytokines and chemokines in TA showed that serum/plasma levels of IL-8, and chemokine (C-C motif) ligands (CCL) 2 and (CCL) 5, cytokines which guide immune cells to the site of inflammation by means of chemotaxis, were increased in TA patients in comparison with healthy controls, and were also elevated when comparing individuals with active TA to those in remission. Consequently, elevated values of pro-inflammatory cytokines, among others CCL2 and 5, in this context may serve as a surrogate parameter for the persisting migration of immune cells to the site of inflammation in affected arteries (37).

Serum IL-6 is considered to be particularly important for assessing disease state and disease activity in TA (37). A presumable significance of IL-6 in the development of TA could be shown by analyzing peripheral blood mononuclear cells (PBMC) from TA patients which produced more IL-6 upon stimulation than their counterparts from individuals with Behçet's disease (37). Apart from that IL-6 was found to be upregulated in inflammatory infiltrates of affected vessels from patients with active TA (37). Monitoring IL-6, which is broadly available and can be performed at relatively low cost, could thus help tailoring therapies to individual patients.

Matrix-metalloproteases might be further suitable markers for distinguishing active and inactive TA (49). Matrix-metalloproteases (MMPs) are enzymes capable of tissue remodeling by modifying the extracellular matrix. Matrix-metalloproteases (MMPs) are found on the surface of immune cells. Consequently, higher MMP levels are required in active inflammatory diseases, since the internal elastic lamina of affected vessels needs to be surmounted in order to allow leukocytes to proceed toward the lamina intima (38).

In addition, low values of the soluble receptor for advanced glycation end products (sRAGE) were suggested as an indicator of active TA (50). sRAGE are membrane spanning receptors and belong to the entity of immunoglobulins (51). Activated cells, e.g., macrophages, release ligands which bind to RAGE, which results in low levels of sRAGE. There are limitations of the mentioned studies: All examinations were performed with specimens from the aorta, limiting potential findings to this part of the vasculature (46). Therefore, a broader range of vessels and cell types should be investigated in future studies.

Similar to GCA, PTX-3 was also found to be involved in TA and might help to distinguish active patients with TA from those in remission (52). One noteworthy point is that an elevation of PTX-3 is not necessarily accompanied by increased CRP levels (53). The concentration of PTX-3 but not that of CRP was, according to Tombetti et al., significantly increased in TA patients who suffered from worsening arterial lesions detected by conventional and computed tomography (CT) angiography. Consequently, plasma PTX-3 levels could serve as a surrogate parameter of disease activity in arteries (48, 52, 54, 55). The reasons for an increase in PTX-3 in TA are similar to those mentioned in the context of GCA. Some authors suggest a local production of PTX-3 in affected vessels by those cells contributing to forming an inflammatory infiltrate (49, 52, 53)).

If these findings can be confirmed in larger cohorts, plasma levels of PTX-3 may in future help to differentiate between active and inactive disease (52).

Nuclear Magnetic Resonance Spectroscopy (NMR) based serum metabolomics has identified distinct profiles in individuals suffering from TA in comparison to age- and sex-matched controls (56). The best discriminatory potential was found for N-acetyl glycoprotein (NAG), an anti-inflammatory metabolite, and glutamate, a neurotransmitter often associated with inflammation (56–58). Upon follow up, changes in metabolic spectra evolved with a change in disease activity. Thus, certain metabolic profiles allow the distinction between clinically active and inactive TA patients, representing potential biomarkers for disease activity and delivering additional information which can serve as a guide to therapy (56). Future studies should further investigate these findings in larger cohorts with a longer follow up duration.

Yilmaz et al. found that plasma levels of unacylated and acylated ghrelin (growth hormone release inducing), a peptide hormone which regulates appetite and energy use, could be of use for intermittently evaluating the activity of TA and identifying the best possible way to treat TA patients. Furthermore, serum leptin levels, with leptin diminishing the feeling of hunger, and the leptin/ghrelin ratio might help to assess disease activity (59). Other studies confirm a bidirectional relationship between Ghrelin, Leptin, and inflammation. (60).

A potential biomarker which is supposed to be informative with regard to the effects of therapy on patients with TA treated with glucocorticoids and tumor necrosis factor-alpha inhibitors was identified by Serra et al. in the form of neutrophil gelatinase-associated lipocalin (NGAL) that modulates the activity of MMP-9 (61). NGAL plasma concentration has been shown to be associated with diseases of the vasculature (62). Furthermore, as mentioned above, MMP levels are known to be increased in inflammation so that changes in NGAL levels can be expected. Apart from the low number of patients an important limitation of the studies is the lack of prospective data.

Further possible biomarkers for TA comprise certain autoantibodies, e.g., anti-ferritin antibodies and anti-endothelial cell antibodies (AECA) (63). Antibodies against Annexin V (AA5A), a factor which contributes to the regulation of (endothelial) cell apoptosis, were identified in a certain percentage of individuals with TA and were associated with AECA, so that AA5A represents another potential biomarker for the management of TA (48, 64). Further data suggest that AECA, which are capable of initiating apoptosis of endothelial cells, are crucially involved in vascular damage observed in TA patients (65–67). The majority of AA5A associated disorders, e.g., systemic lupus erythematosus, and rheumatoid arthritis exhibit AECA as well (64, 68–73). Therefore, AA5A has been proposed as one of the antigen targets of AECA (64). Nonetheless, the actual antigens of AECA have not yet been identified. The differences between isotypes of AECA need to be elucidated in the future. Furthermore, studies on the factors triggering the formation of AA5A and their role under physiological and pathological conditions are required (64).

Dogan et al. focused on markers of destructive and reconstructive processes of the endothelium, such as circulating endothelial cells (CEC), circulating endothelial progenitor cells (CEPC) and vascular endothelial growth factor (VEGF) (49). According to the corresponding study, elevated levels of CEC were detected in patients with TA. Besides, CEC levels showed a slightly positive correlation with CRP levels. Only CEPC and VEGF levels turned out to assume higher values in active compared to inactive patients. Thus, these two markers may correlate with disease activity (74). These findings are based on the following considerations: Systemic vasculitis is, among others, due to endothelial injury (75, 76). This injury causes the detachment of mature endothelial cells, called CEC (74). CEPCs are required for tissue repair in response to various cytokines and growth factors, e.g., VEGF (74).

A limitation of the presented study is that treatment effects on these parameters have not been evaluated. Further larger and prospective studies are required to verify the generated data.

Further promising findings were reported by Goel et al. (77). At the baseline assessment of a longitudinal single center study, median serum myeloid-related protein 8/14 (MRP 8/14) levels, also known as calprotectin, was found to be increased in subjects suffering from TA compared to healthy subjects. In addition, active disease states were characterized by higher MRP 8/14 levels when compared to stable disease states or healthy controls. MRP 8/14 is part of a group of cytosolic calcium-binding protein family called S100 (78). During the course of disease, changes in serial MRP 8/14 levels were linked to disease activity, no matter what the administered glucocorticoid dose has been (77), so that MRP 8/14 has been proposed as another prognostic biomarker in TA (77).

Finally, Nair et al. identified serum amyloid A (SAA), an apolipoprotein which belongs to the group of acute phase proteins, as a biomarker to evaluate disease activity and treatment response in TA (79). At baseline, there were higher SAA levels in individuals with TA than in healthy controls, and also higher values in patients with active disease compared to those with stable disease. SAA exhibited a decreasing trend during follow-up in treatment responders, whereas these changes were not observed in non-responders (79). The study was limited by small sample size and a short duration of follow up (79).

Taking all the above considerations into account, we conclude that IL-6 is particularly valuable for assessing disease state and disease activity in TA. For a summary of biomarkers see Tables 1, 2.

## Medium vessel vasculitis

### Polyarteritis Nodosa (PAN)

Polyarteritis nodosa exclusively affects medium-sized vessels and is potentially associated with Hepatitis B and C virus (4). In contrast to the aforementioned vasculitides of large vessels PAN is the scarcest type of vasculitis in central Europe. General symptoms include fever, stomach, muscle, and joint pains, and myocardial infarction even in young patients. With regard to neurological complaints, peripheral neuropathy belongs to the most frequent organ manifestations, mostly in the form of mononeuritis multiplex. Also, symmetrical sensorimotor polyneuropathies or an affection of nerve plexi may occur (80). A combination of polyneuropathy in conjunction with Livedo reticularis is highly suspicious of PAN. CNS manifestations in PAN include ischemic stroke, intracerebral and subarachnoid hemorrhage and seizures (4). It has been demonstrated that recessive loss-of-function mutations affecting the gene which encodes adenosine deaminase 2 (ADA2) might lead to polyarteritis nodosa vasculopathy (81).

As far as the pathogenesis of the disease is concerned Shimojima et al. showed an expansion of T-helper cells and an impaired function of regulatory T cells (82). These findings suggest that PAN results, among others, from defects in T-cell-mediated immunity (82). The changes might be due to insufficient suppressive abilities of regulatory T cells with regard to CD4^+^ T cells and an impaired cytotoxic T-lymphocyte-associated Protein 4- (CTLA-4-) expression of regulatory T cells. CTLA-4 is essential for the inhibition of the proliferation of CD4^+^ T cells, i.e., T-helper cells. In order to better understand the underlying pathomechanisms, larger cohorts need to be examined.

Manolov et al. found higher levels of plasma VEGF (see remarks on TA) in subjects with vasculitic neuropathy compared to healthy controls (65). This is why plasma VEGF levels could be of use in predicting vasculitic neuropathy (65). However, this finding does not only apply to PAN and the number of included patients was extremely low so that the current practical benefit of this biomarker is very limited.

In PAN patients with cutaneous manifestations, Okano et al. suggested that anti-phosphatidylserine-prothrombin complex (anti-PSPT) antibodies could be regarded as a biomarker for the existence of PAN and therefore support making an early diagnosis (83). These antibodies turned out to decrease to lower levels after treatment with cyclophosphamide and glucocorticoids in comparison with the pre-treatment levels (83). Mechanistically, it is assumed that prothrombin binds to apoptotic endothelial cells and phosphatidylserine (83). The resulting complexes are thought to trigger the production of anti-PSPT antibodies resulting in the development of PAN (83). Apart from that, deteriorations of the clinical disease course might be associated with higher levels of anti-moesin-antibodies. Moesin is an intracellular protein connecting cell membrane and cytoskeleton (83). Anti-moesin antibodies have been suspected to exacerbate PAN via antiphospholipid antibodies, such as anti-PSPT antibodies (83). However, these findings need to be validated in a prospective study.

There have been few studies on biomarkers in polyarteritis nodosa. Autoantibodies against lysosomal-associated membrane protein-2 (LAMP-2) showed an association with dermatological signs of the disease, but in the end there was no significant difference when compared to values measured in healthy controls (66). LAMP-2 is a component of the lysosomal membrane. It has been assumed that LAMP-2 plays a role in the pathogenesis of vasculitis (66). Mechanistically, Takeuchi et al. hypothesize that anti-LAMP-2-antibodies bind to neutrophils, which then infiltrate small vessels of the skin (66). The main limitation of the study consists in the fact that the association with LAMP-2-antibodies seems to exclusively refer to cutaneous manifestation.

To sum up, the tested biomarkers either refer to cutaneous manifestations or bear the potential to predict vasculitic neuropathy, not only in PAN. With regard to the latter aspect, the corresponding study design does not allow to call VEGF an established biomarker. For a summary of biomarkers see Tables 1, 2.

## Small vessel vasculitis

Granulomatosis with polyangiitis (GPA), Microscopic polyangiitis (MPA), and eosinophilic granulomatosis with polyangiitis (EGPA), previously known as Churg-Strauss syndrome, form the group of ANCA (anti-neutrophil cytoplasm antibody)-associated vasculitides (AAV). The AAV, as well as cryoglobulinemic vasculitis, Ig A vasculitis, and Behçet's disease, potentially affecting all types of blood vessels, will be considered in this chapter.

Before focusing on certain subtypes, biomarkers relevant for AAV in general are reviewed: GPA and MPA patients in remission were shown to have higher levels of CD25^+^ regulatory B cells in proportion to individuals with active vasculitis (67). CD25 is the α-subunit of the Interleukin-2-receptor, which influences the binding process between receptor and ligand. CD25^+^ B cells are therefore supposed to have regulatory functions in the mentioned vasculitides (67). To confirm or disprove the role of regulatory B cells as a marker of AAV these shortcomings should be overcome in future investigations.

Effective ways to predict relapse rates have been discovered in both GPA and microscopic polyangiitis. First, anti-MPO positive patients showed lower relapse rates than anti-PR3 positive subjects (84–86). Second, a specific gene expression signature in CD8^+^ cells was identified, which seems to predict relapses (87): Transcriptional profiling analysis has revealed that CD8^+^ T cell expression may represent a tool to divide patients into two distinct subgroups (relapse/active disease vs. remission/stable disease) (87, 88). The group of patients with a poor prognosis was characterized by a subset of genes playing a role in the IL-7 receptor pathway and T cell receptor signaling. The CD8^+^ T cell memory cell population, which has been suspected to promote disease relapses, was also expanded in this group. A possible explanation of these observations could be that altered gene expression in naïve CD 8^+^ T cells might be the result of genetic modifications in the phase of T cell maturation in the thymus (87, 89–91). As a reaction to antigen exposure, this could lead to T cell proliferation, at least in part via signaling cascades involving IL-7 receptor and T cell receptor, resulting in an increasing number of memory cells (92, 93). In order to use this information to individualize the therapy in AAV depending on a patient's classification as “active” or “stable,” a prospective clinical study is required.

Moon et al. identified the CRP to serum albumin ratio (CAR) as an independent predictor of all-cause mortality in patients suffering from AAV with a similar potency as diabetes mellitus (94). Notably, the combined occurrence of CAR ≥ 10.35 and diabetes mellitus had a higher mortality, regardless of the actual reason, than the lack of these characteristics (94). These observations might have the following reasons: Chronic inflammation leads to increased IL-6 release, which increases the levels of CRP in the peripheral circulation” (94, 95). Given that IL-6 also diminishes the hepatic albumin production, the CAR is altered in a specific manner (94). The limitations of this study consist in the small sample size derived from a single center, its retrospective character, and the lack of a clear cut-off value of CAR to predict all-cause mortality in patients with AAV (94).

In AAV patients, myeloperoxidase-(MPO-)ANCA levels returned to values within normal limits during a period of 6 months after the initiation of remission induction therapy. A reappearance of MPO-ANCA could be detected in 40% of these individuals and occurred more often in relapsing subjects than in age- and sex-matched non-relapsing controls (96). Therefore, the reappearance of MPO-ANCA might be a biomarker which bears the potential to predict a relapse in individuals “with MPO-ANCA-positive AAV in” remission (96). Becoming ANCA-negative before maintenance therapy has been initiated was connected to a reduced risk of relapse (96). The frequency of relapses in general is much higher in PR3-ANCA- than in MPO-ANCA-positive disease, whereas there is a reverse relation with regard to mortality.

The so called delta neutrophil index (DNI), a simultaneous view at the number of immature granulocytes and the consumption of neutrophil granulocytes, correlates with the Birmingham vasculitis activity score at diagnosis—an instrument which helps assessing disease activity in patients with PSV (97). DNI values ≥0.65% were shown to be a surrogate parameter for a difficult progression of AAV and relapse in GPA and MPA during the observation period (97). This connection may be the result of the following considerations: T cells and macrophages release inflammatory cytokines that prime neutrophils and cause an increase in adhesion molecules on neutrophils and endothelial cells. ANCA promote the interaction of neutrophils and endothelial cells, which results in the diapedesis of neutrophils through the vascular cell layers. Finally, activated neutrophils induce vasculitis by reactive oxygen radicals and degranulation (97–104).

Another potential biomarker for disease activity is the tissue inhibitor of metalloproteinase 1 (TIMP1). TIMP1 is responsible for the regulation of MMPs (105–107). In accordance with MMPs, TIMP1-levels increase in inflammatory states. Additionally, unlike CRP, TIMP1-levels were significantly higher in individuals suffering from active AAV than in infected subjects without disease activity (108). Therefore, TIMP1 can be used to distinguish between AAV with mild activity and remission.

Autoantigen gene loci in ANCA-associated vasculitis Proteinase 3 (PRTN3) and MPO were found to undergo epigenetic modifications, which correlated with disease activity and with PR3 and MPO expression. Epigenetic changes in general may comprise DNA methylation, histone modifications, and non-coding RNA (109–111). Thus, it appears that not only mutations, i.e., changes in the DNA sequence, may have far-reaching consequences for the manifestation of certain diseases. It still has to be elucidated which factors cause certain epigenetic modifications resulting in AAV and, maybe, how they can be avoided or prevented.

Further biomarkers for different types of AAV are, among others, distinct cytokine profiles of PR3-AAV, i.e., IL-6, granulocyte-macrophage colony-stimulating factor (GM-CSF), IL-15, IL-18, CXCL (C-X-C motif chemokine) 8/IL-8, CCL-17/thymus and activation-regulated chemokine, IL-18 binding protein, soluble IL-2 receptor α (sIL-2Rα), and nerve growth factor ß” (NGF-ß). Cytokine profiles of MPO-AAV comprise soluble IL-6 receptor, soluble tumor necrosis factor receptor type II (sTNFRII), neutrophil gelatinase-associated lipocalin (NGAL), and soluble intercellular adhesion molecule 1. Osteopontin, sTNFRII, and NGAL can be attributed to MPA, whereas IL-6, GM-CSF, IL-15, IL-18, sIL-2Rα, and NGF-ß can be attributed to GPA (112). Although these biomarkers bear the potential to improve the handling of ANCA-associated vasculitides, not all of them are suitable for everyday use.

Data derived from the RAVE (Rituximab in ANCA-Associated Vasculitis) trial allow the following conclusions: Patients treated with rituximab, who were anti-PR3 positive and exhibited an increase of calprotectin serum levels between the start of data collection and month 2 or month 6, exhibited a higher risk of relapse at 18 months. Hence, serum calprotectin might guide more intensive or prolonged treatment (113).

Furthermore, anti-PTX3 antibodies were frequently detected in AAV patients and antibody levels were increased in comparison to healthy controls. Comparing the states of remission and active disease, anti-PTX3 antibody values turned out to be higher in the latter case (114). In summary, anti-PTX3 antibody levels allow to distinguish between differential diagnoses and to evaluate disease activity.

Simon et al. hypothesize that Anti-PTX3 autoantibodies attract PTX3-expressing apoptotic neutrophils and enhance their phagocytosis (114). The interaction of PR3- and MPO- expressing neutrophils and ANCA induces phagocytosis and the synthesis of pro-inflammatory cytokines (114–116). Additionally, PTX3 can also be found on the surface of endothelial cells, which, in case of endothelial cell injury, may attract immune cells (114). It still remains to be examined whether the immunogenic capacity of apoptotic cells depends on anti-PTX3 and in how far there is an effect on disease duration (114).

Among others, DNI and CAR seem to be promising, but further studies are required to validate existing findings. For a summary of biomarkers see Tables 1, 2.

### Granulomatosis With Polyangiitis (GPA)

GPA is a rare form of small vessel vasculitis with an incidence rate of 0.9/100,000/year that affects both sexes equally (4). In the early stage, local inflammatory processes accompanied by granuloma formation take place in the respiratory tract and later on the disease spreads in terms of a generalization into small vessel vasculitis (117, 118). Leading symptoms in the early stadium of GPA is a bloody and barky nasal discharge. As soon as GPA generalizes, the spectrum of potential manifestations includes general symptoms and an affection of the lungs (therapy-resistant pneumonia with coughing, dyspnea, and hemoptysis), the kidneys in terms of glomerulonephritis, the skin in the form of various efflorescences, the eyes (conjunctivitis, episcleritis), the heart (pericarditis, vasculitis of the coronary arteries, cardiomyopathy), and the nervous system. Furthermore, a saddle nose is a symptom of advanced GPA. The latter can be affected by peripheral neuropathies, polyneuropathies, as well as mononeuritis multiplex. These peripheral neuropathies can be the initial disease manifestation of GPA and of other AAV (119). Therefore, neuropathies of unknown etiology should be inspected for signs of AAV, especially when typical stigmata of AAV are present. In AAV patients, peripheral neuropathy manifestation is associated with a higher number of affected organs, an increased ANCA-titer and a more severe disease course compared to individuals without peripheral neuropathy (120, 121). Cerebral small vessel vasculitis is a rather scarce phenomenon in these patients. Additionally, GPA patients might suffer from pachymeningitis and granulomas of the pituitary gland. GPA is associated with ANCA, above all PR3-ANCA, in 50% of cases in the early, local stadium, and in at least 90% of cases in a later, more generalized stage (122–124).

Biomarkers already in use for assessing disease activity and predicting flares are ESR, CRP, anti-PR3, and anti-MPO titers but, as already mentioned, their informative value is limited, since they are much less useful in evaluating the activity of GPA in individuals, in whom a diagnosis has already been made (3). So far, useful markers of inflammation, angiogenesis, tissue damage, and repair have been identified. Observing MMP-3, TIMP-1, and CXCL13 indicated significant differences between groups of patients. Nonetheless, due to the poor correlation of the markers among each other it has been suggested that a combination of these biomarkers might be the most promising approach (125–127). CXCL13 is a chemokine that attracts immune cells to the site of inflammation. The reasons for increased CXCL13 levels are not fully understood, but high CXCL13 levels have been linked to B cell malfunction (127). Furthermore, sRAGE are considered to mirror the cumulative burden of granulomatous inflammation in GPA, so that sRAGE may in future be established as a mean of assessing disease activity (128).

Further studies are required to detect the changes of sRAGE over time with treatment and exacerbations of the disease.

In patients who receive rituximab, the simultaneous consideration of B cell reconstitution after depletion due to the therapy and ANCA-PR3 levels could fulfill the function of a biomarker for a GPA relapse, given that Cartin-Ceba et al. observed that all occurring relapses in the cohort under investigation were accompanied by an increase in B cells, preceded by an increase in ANCA-PR3 levels (129) (for a summary of biomarkers, see Tables 1, 2).

Hitherto there are no directly applicable biomarkers in GPA.

### Microscopic Polyangiitis (MPA)

Microscopic polyangiitis (MPA) shares some characteristics with GPA regarding the clinical manifestations. Being a necrotizing inflammatory process of small vessels it mostly affects the kidneys and, less often, the lungs, the skin, and the nervous system (4). The latter is mainly affected by peripheral neuropathy, mostly mononeuritis multiplex, by intracranial hemorrhage, seizures, headache and cerebral vasculitis. MPA is strongly associated with ANCA, especially (myeloperoxidase) MPO-ANCA, in 75% of all cases.

Saito et al. demonstrated that carbonic anhydrase III (CAIII) antibodies had a significantly higher prevalence in MPA patients in comparison to healthy controls. Additionally, the authors report, especially in proportion to antibody-negative subjects, elevated values of activity scores for vasculitic disorders in those cases of MPA which came along with anti-CAIII antibodies (130). Therefore, these antibodies might facilitate diagnosing MPA in ANCA-negative patients (70% of MPA patients are pANCA positive) and for assessing disease activity (130). Carbonic anhydrases are zink metalloenzymes that catalyze the hydration of carbondioxide and are present in red skeletal muscle. It is suggested that CA III plays a role in intracellular signaling, particularly in response to oxidative stress (131, 132). Possibly, anti-CAIII-antibodies protect cells from oxidative damage during inflammation (130). The informative value of the results is limited to the low number of patients, highlighting the necessity of future studies involving a higher number of patients.

Hitherto there are no directly applicable biomarkers in MPA.

### Eosinophilic Granulomatosis With Polyangiitis (EGPA)

Eosinophilic granulomatosis with polyangiitis (EGPA), previously known as Churg-Strauss syndrome, can be briefly characterized as a granulomatous, necrotizing vasculitis with eosinophilia that is accompanied by pulmonary manifestation with severe asthma attacks as the leading symptom. An ANCA-association, usually MPO-ANCA, is given in 40% of all cases (133). Nervous system manifestations comprise vasculitis of the central nervous system with impaired consciousness as well as sensorimotor deficits. Moreover, EGPA may result in allergic rhinitis/sinusitis, myocarditis, and purpura.

After the initiation of treatment, eosinophilia, the hallmark of EGPA, is not an appropriate biomarker for disease activity, since the eosinophilic cell count tends to drop rapidly as soon as glucocorticoids are administered (134). It has been demonstrated that proteins related to eosinophilia, first of all eoxtaxin-3, and to Th2 immune response in general, particularly IgG4 and CCL17/TARC, were elevated in active EGPA. This elevation was also found when EGPA was compared to inactive disease, other diseases characterized by vasculitis or hypereosinophilia, and healthy controls (135–138). Eotaxins are chemokines which contribute to the accumulation and maturation of eosinophils (136). CCL17/TARC is a chemokine that is released by dendritic and endothelial cells. It is involved in the targeted attraction of activated Th2 lymphocytes to affected foci (135). Further investigations are necessary to increase knowledge on additional characteristics of eotaxin-producing cells in EGPA (136). Higher IgG4 levels might be caused by activated Th2 lymphocytes but the actual pathological role of IgG4 in EGPA is still unclear.

In a cohort of refractory and relapsing EGPA patients treated with rituximab, individuals exhibiting ANCA had a better chance to reach the state of remission (139).

Eotaxin-3, IgG4, and CCL17/TARC are promising future biomarkers, but they are not completely understood yet. For a summary of biomarkers, see Tables 1, 2.

### Cryoglobulinemic Vasculitis (CV)

Cryoglobulinemic vasculitis (CV) is a rare disease affecting primarily small vessels due to the formation of immune-complexes, and is accompanied by Hepatitis C virus infection in the large majority of all cases. The main symptoms are purpura, ulcerations, arthritis, and, as a late symptom or in severe courses, glomerulonephritis. With regard to neurological affliction, patients have polyneuropathy and less frequently focal neurological signs resulting from cerebral manifestations (140). Cryoglobulinemic vasculitis is mainly characterized by the combination of arthritis, purpura, and polyneuropathy, in conjunction with complement consumption (low C4 levels) and the evidence of rheumatoid factor (141).

Monoclonal rheumatoid factors (mRF) carrying the WA cross-idiotype (Xid) are of major importance in the pathogenesis of cryoglobulinemic vasculitis in individuals with hepatitis C virus (HCV) infection (142). When the WA Xid is found, individuals without symptoms but with HCV infection can be shown to exhibit WA B cells (142). Consequently, WA B cells in asymptomatic individuals with HCV infection might represent a marker for the development of CV (142). Furthermore, B-cells expressing VH1-69 were considered to be the target of treatment of infectious CV. Therefore, using this cell population as a biomarker might provide information on the activity of CV and response to treatment. A possible future perspective consists in the development of an anti-WA Xid antibody as a method for everyday medical use (142). Maybe novel treatment strategies can be derived from such antibodies.

A further study showed that patients with HCV-related mixed cryoglobulinemia (HCV-MC) exhibited significantly higher mean IL-6 values compared to healthy controls and individuals suffering from HCV chronic hepatitis without concomitant cryoglobulinemia (HCV^+^). IL-6 was also elevated in cryoglobulinemic subjects with active vasculitis, thus representing potential indicator for this state (143). A hallmark of HCV-MC is the synthesis of immune-complexes, primarily cryoglobulins incorporating HCV, evoking vasculitis (143). As a consequence, IL-6 levels rise and enhance inflammation (143). However, the prognostic significance of IL-6 in HCV-MC needs to be examined in larger prospective studies (143).

Moreover, increased values of CXCL10 and TNF-alpha were measured in individuals with HCV-associated cryoglobulinemia (144). In HCV-MC patients, active vasculitis was associated with increased CXCL10 values (144). C-X-C motif chemokine 10 (CXCL10), also known as Interferon gamma-induced protein 10 (IP-10) or small inducible cytokine B10, is a chemokine relevant for chemoattraction of immune cells (144). It is hypothesized that HCV induces the production of CXCL10, which in turn contributes to the development of chronic hepatitis C (144, 145). Further investigations comprising higher numbers of patients will have to test the potential of CXCL10 to predict the disease course of HCV-MC patients and guide the treatment (144).

Hitherto there are no clinically proven biomarkers. For a summary of biomarkers see Tables 1, 2.

### Ig A vasculitis

Ig A vasculitis, a vasculitis of small vessels driven by immune complexes, represents the most common vasculitis in childhood (4). Immunoglobulin A (IgA) plays a pivotal role in the disease. Typical clinical manifestations are IgA-nephritis, gastrointestinal symptoms such as stomach pain and hematochezia, purpura, and nephritis. The disease can be associated with cerebral ischemia, intracranial hemorrhage, diffuse cerebral edema, peripheral neuropathies, facial nerve paresis, and Guillain-Barré syndrome (146). Neurological complaints, however, are a rather scarce manifestation of Ig A vasculitis (147).

In pediatric patients with Ig A vasculitis, white blood cells (WBC), serum levels of CRP, IL-6, and SAA (amyloid A) showed a significant increase compared to healthy individuals (148). In addition, a complex serum biomarker index (SAA+IgA/4000+IgM/4000x0.4CRPmeanvalueCRPi) is significantly higher in HSP patients compared to healthy controls (148). The discrimination of these states is enabled by the integration of biomarkers specific and sensitive for Ig A vasculitis, such as SAA, and other biomarkers also connected to inflammatory processes in general, i.e., CRP. The biomarker index can be easily calculated and the number of patients exceeded 100 individuals so that it seems ready for use in discriminating patients with Ig A vasculitis and septicemia or the absence of one of these disorders.

High performance liquid mass spectrometry (HPLC-MS) revealed that levels of fecal secondary colonic bile acids, deoxycholic acid, and lithocholic acid are significantly lower in children with Ig A vasculitis in the acute stage and in remission than in healthy controls (149) (for a summary of biomarkers, see Table 1).

The biomarker index is an already available tool that can be used in the treatment of patients with IgA vasculitis.

### Behçet's Disease (BD)

Behçet's disease (BD) differs from the aforementioned vasculitides, since both arterial and venous blood vessels of any diameter can be affected. There is a strong association with human leukocyte antigen (HLA) B51 and the leading symptom consists of oral and genital aphthae (4). The peripheral nervous system is rarely affected by polyneuropathies. Manifestations in the central nervous system are divided into a parenchymatic and a non-parenchymatic neurovascular type. The parenchymatic type is characterized by bihemispheric lesions which can cause hemiparesis, hemihypesthesia, vision field losses, seizures, movement disorders, speech disorder, and Parkinson-like symptoms. Furthermore, aseptic meningitis and affections of the brain stem with cranial nerve paralysis, sensorimotor disturbances, and cerebellar symptoms may occur (150–153). The neurovascular type of BD comprises sinus vein thrombosis and increased cerebral pressure, resulting in headache, focal neurological signs, seizures, papilledema, impaired consciousness, and paralysis of the sixth cranial nerve. The affection of arteries may lead to aneurysms, dissections, and consecutive intracranial hemorrhage or cerebral infarction. Peripheral neurological manifestation may consist of Guillain-Barré syndrome, polyneuropathy, autonomous neuropathy, and mononeuritis multiplex (150–153).

In a genome wide association study, the central role of HLA-B51 in regard to BD susceptibility has been confirmed (154). Apart from the connection to HLA-A and HLA-C, BD susceptibility was also associated with cytokines and danger signals IL-10, IL-23R, CCR1, STAT4, KLRC4, GIMAP2/GIMAP4, and UBAC2 genes. These findings hint at an impaired response to danger signals of the human organism and impaired immunological processes in BD patients (154). The presence of HLA-B51 is known to be accompanied by a marked increase in the risk of developing Behcet's disease, but the underlying pathophysiologic mechanisms have not been fully elaborated (154). Case-control studies with higher number of participants could help to elucidate connections between genetic attributes and certain phenotypes of BD (154).

The fact that not only blood but also other body fluids can contribute to the development of new biomarkers was underlined by Ahn et al, who distinguished BD patients from healthy individuals by employing a group of 10 urine derived metabolites, i.e., guanine, pyrrole-2-carboxylate, 3-hydroxypyridine, mannose, l-citrulline, galactonate, isothreonate, sedoheptulose, hypoxanthine, and gluconic acid lactone (155). Similarly, a panel of five metabolic markers derived from serum, i.e., decanoic acid, fructose, tagatose, linoleic acid, and oleic acid, were found to be potential biomarkers of BD with a sensitivity of 100% and a specificity of 97.1% (156). Future studies should include larger sample sizes in order to confirm the results. Apart from that, future studies should take the circadian rhythm of certain metabolites and their dependence on other factors, such as physical activity, into consideration (156).

The expression of Tissue Factor (TF) by microparticles was experimentally shown to contribute to the development of thrombosis (157). Using flow cytometry, it has been demonstrated that the total number of plasma microparticles was elevated in BD patients compared to healthy controls, as were microparticles characterized by TF and Tissue Factor Pathway Inhibitor (TFPI) (157). BD patients who had already presented with thrombosis in the past exhibited elevated counts of total and TF positive microparticles in comparison to individuals without thrombosis, but showed a lower percentage of TFPI positive microparticles. In conclusion, microparticles characterized by TF are elevated in BD and a discrepancy or impaired balance between TF and TFPI could represent a risk factor for thrombosis (157). In order to improve the knowledge about the usefulness of these markers, larger and prospective studies, e.g., in endemic regions, are required.

Yoshioka et al. analyzed protein profiles of peripheral blood mononuclear cells (PBMCs) and compared them to profiles from patients with rheumatoid arthritis (RA) and Crohn's disease (CD), and to healthy controls (HC) (158). Tyrosine-protein phosphatase non-receptor type 4, threonine synthase-like 2, and ß-actin were identified as possible biomarker candidates for BD, since they can be applied to discriminate inflammatory bowel disease from BD and other diseases (158). Tyrosine-protein phosphatase non-receptor type 4 binds to cytoskeletal proteins and is involved in cellular responses to cytokines. Threonine synthase-like 2 exacerbates inflammation, can act as a cytokine and can promote the production of IL-6 in osteoblasts (158, 159). ß-actin is a structural protein which represents a part of the sarcomere of muscle fibers. The changes in ß-actin levels may be due to structural changes in the actin cytoskeleton as soon as T cells are activated in BD (158). In order to learn more about the significance of the identified proteins, the analysis of the protein expression of different kinds of immune cells may be of interest with regard to the pathophysiological mechanisms underlying BD. By doing so, novel biomarkers for everyday use could be developed.

Sota et al. reported that serum amyloid-A (SAA) levels can be useful to predict the affection of organs and repetitive affections of the eyes, but they also found that SAA cannot serve as a surrogate parameter for disease activity (160). SAA plays a role in the acute phase reaction and is frequently elevated in autoimmune disorders. Some authors suggest that SAA interacts with certain cytokines promoting inflammation, e.g., IL-1ß, which might be involved in the development of BD (161, 162). Future studies should be designed as prospective investigations, including a control group because these features are lacking in Sota's study.

A further study identified potential antigens in circulating immune complexes of BD patients but not in those of healthy subjects. Tubulin-α-1c is such an antigen and antibodies directed against it were increased in individuals with BD in comparison to healthy controls and disease controls (systemic lupus erythematosus, recurrent aphthous ulcers, AAV, and TA). In diagnosing BD, the tubulin-α-1c antibody had a sensitivity of 61.4% and a specificity of 88.4% (163). Additionally, an association of anti-tubulin-α-1c with deep venous thrombosis and erythema nodosum, and a significant correlation with the extent of inflammatory processes in BD and indicators of disease activity, such as ESR, CRP and BVAS (Birmingham Vasculitis Activity Score) were found (163). As part of the cytoskeleton, tubulins maintain the shape and structure of cells, GTPase activity and intracellular movement (163). Tubulin-α-1c was moreover shown to increase VEGF levels and cause endothelial damage under vasculitic conditions and in cases of thrombosis (163). However, precise mechanisms have not been elaborated.

As far as the assessment of disease activity in BD is concerned, a substantial gain in information resulted from the analysis of the platelet to lymphocyte ratio (PLR) and the lymphocyte to monocyte ratio (LMR). The PLR was remarkably higher in BD patients, whereas the LMR assumed lower values in subjects with BD compared to controls (164). When comparing active and inactive BD, active BD was characterized by significantly higher PLR, ESR and highly sensitive CRP. Furthermore, PLR and LMR were demonstrated to be independent factors for BD (164). To sum up, PLR may be useful as a novel biomarker to evaluate disease activity in BD which is not expensive but can be easily used. Current data derives from a cross-sectional analysis in a single center. Future studies should be performed in multiple centers over a longer period of time. Beyond, treatment effects should also be evaluated (164).

In summary, there are not no sufficiently tested and established biomarkers for BD apart from HLA B51. For a summary of biomarkers see Tables 1, 2.

## Conclusion

Biomarkers are of importance in improving the diagnostic process, the treatment, and the prognosis for patients suffering from various forms of vasculitis. Nonetheless, the majority of the findings reviewed in this article do not exclusively apply to neurological manifestations of vasculitides. Further studies that employ appropriate control groups, use precise definitions for disease states such as “active” and “remission,” and clearly outline the type of neurological manifestation involved are needed to guide our use of biomarkers in vasculitides of the nervous system.

## Author Contributions

DS conceptualized and wrote the manuscript. AS-P contributed to writing the manuscript. CB, LM, CK, and SM gave valuable suggestions for improving the manuscript. JM conceptualized the manuscript and contributed to writing the manuscript.

### Conflict of Interest Statement

SM has received honoraria for lecturing, travel expenses for attending meetings and financial research support from Almirall, Bayer Health Care, Biogen, Diamed, Genzyme, MedDay Pharmaceuticals, Merck Serono, Novartis, Novo Nordisk, ONO Pharma, Roche, Sanofi-Aventis, Chugai Pharma, QuintilesIMS and Teva. JM has received grants from Deutsche Forschungsgemeinschaft, Bundesministerium für Bildung und Forschung (BMBF), Else Kröner-Fresenius-Stiftung, EVER Pharma Jena GmbH, Ferrer International, travel grants from Boehringer Ingelheim and speaking fees from Bayer Vital. The remaining authors declare that the research was conducted in the absence of any commercial or financial relationships that could be construed as a potential conflict of interest.
